# Sensory block level prediction of spinal anaesthesia with 0.5% hyperbaric bupivacaine: a retrospective study

**DOI:** 10.1038/s41598-021-88726-2

**Published:** 2021-04-27

**Authors:** Yu-Yin Huang, Kuang-Yi Chang

**Affiliations:** 1 Department of Anesthesiology, Cheng Hsin General Hospital, Taipei, Taiwan; 2 Department of Biomedical Engineering, Ming Chuan University, Taoyuan, Taiwan; 3 Department of Anesthesiology, Taipei Veterans General Hospital, Taipei, Taiwan; 4 School of Medicine, National Yang-Ming University, Taipei, Taiwan

**Keywords:** Pain management, Therapeutics, Surgery

## Abstract

There is still no consensus on how to determine the dose of spinal anaesthesia with adequate sensory block for a planned surgery. This retrospective study aimed to explore the associations of miscellaneous factors with peak sensory block level after spinal anaesthesia with hyperbaric bupivacaine, and to construct a predictive model for single-shot spinal anaesthesia. We collected the records of 401 non-pregnant adults who underwent spinal anaesthesia with 0.5% hyperbaric bupivacaine at the L3–4 or L4–5 intervertebral space for lower body surgeries. Multiple linear regression analysis was used to investigate predictors of the block level and build up the predictive model. Five variables were identified as independent predictors of the peak sensory block level, including bupivacaine dose, height, weight, gender and age. The predictive model for peak block level after spinal anaesthesia could be expressed as a formula with these five variables and the estimated predictive power was 0.72. Based on this model, it is possible to determine a reasonable dose of hyperbaric bupivacaine for spinal anaesthesia, which gives adequate sensory block required for diverse surgical procedures in various patients and could be considered as a dose reference for sensory block height in spinal anaesthesia.

## Introduction

For more than one hundred years, spinal anaesthesia has been used as a simple, fast and reliable technique in a wide range of lower body surgeries^1^. In addition to sensory nerve blockade, it also causes simultaneous autonomic and motor inhibition. Much higher cephalad spread could lead to sympathectomy-induced hemodynamic instability along with nausea and vomiting as well as shortness of breath caused by abdominal or intercostal muscle weakness. Conversely, much lower block height may not satisfy the surgical demand and may require conversion to general anaesthesia during an ongoing operation^2,3^. Although mounting evidence over a number of decades has revealed there are a number of factors that may influence intrathecal anaesthetic spread, including the contents of the injected solutions, clinical procedures, patient variables and so on^4,5^, how to predict sensory block height after spinal anaesthesia remains an unresolved clinical issue.

In our long-term daily practice, greater cephalad spread of sensory block appears to be observed in patients who are shorter, overweight, of female gender, older or who have been administered higher doses of local anaesthetic, such as hyperbaric bupivacaine (heavy Marcaine®), which we routinely use. While previous consensus supports the theory that each of these factors may influence the block level of spinal anaesthesia^4,5^, few studies evaluated their combined effects^6–9^.

Therefore, in this retrospective study, we aimed to explore common factors associated with the sensory block level after spinal anaesthesia, the outcome of interest, and evaluated their individual and combinatorial effects on block height. In addition, a predictive model for dermatomal block level after single-shot spinal anaesthesia using hyperbaric bupivacaine was also developed based on identified influential factors.

## Results

A total of 401 eligible patients received spinal anaesthesia for lower body surgeries and their characteristics are summarized in Table 1. On average, the peak block level was 16 segments from S5 (T7) and the mean dose of hyperbaric bupivacaine was 9 mg.

**Table 1:** Patient demographics.

Characteristics	(Count) Mean	(%) SD	Range
**Sex**			
Female	(187)	(46.6)	–
Male	(214)	(53.4)	–
Age (years)	58	18	20–99
Height (cm)	162	10	132–186
Weight (kg)	68	15	36–119
BMI	25.9	4.5	16.6–40
Heavy Marcaine dosage (mg)	9	3	2.5–16
Peak level	16 (T7)	4	5–22 (S1–T1)

SD, standard deviation; BMI, body mass index.

Simple linear regression analysis revealed a positive correlation between peak block height and the hyperbaric bupivacaine dosage, as well as a negative association between peak block level and body height (Table 2). The association between peak block height and body weight was not significant in the univariate analysis. Female patients and those aged between 75 and 85 tended to have a higher peak block level. It’s worth noting that the hyperbaric bupivacaine dose alone accounted for more than half of the total variances in peak block height (*R*^2^ = 0.51). In contrast, other covariates were responsible for less than 10% of variances in peak block height in the univariate analysis (Table 2).

**Table 2:** Univariate effect of collected variables on peak block level.

Characteristic	*β*	SE	Standardized *β*	*p*	*R*^2^	Adjusted *R*^2^
Bupivacaine dose	0.81	0.04	0.71	< 0.001	0.506	0.505
Height	− 0.10	0.02	− 0.30	< 0.001	0.092	0.090
Weight	− 0.01	0.01	− 0.06	0.242	0.003	0.001
Sex (Female vs. male)	1.95	0.33	0.28	< 0.001	0.077	0.075
**Age**					0.019	0.015
76–85 vs. ≤ 75	1.26	0.47	0.13	0.008		
> 85 vs. ≤ 75	1.12	0.78	0.07	0.152		

*β*, regression coefficient; SE, standard error; *R*^2^, coefficients of determination.

**Figure 1:**
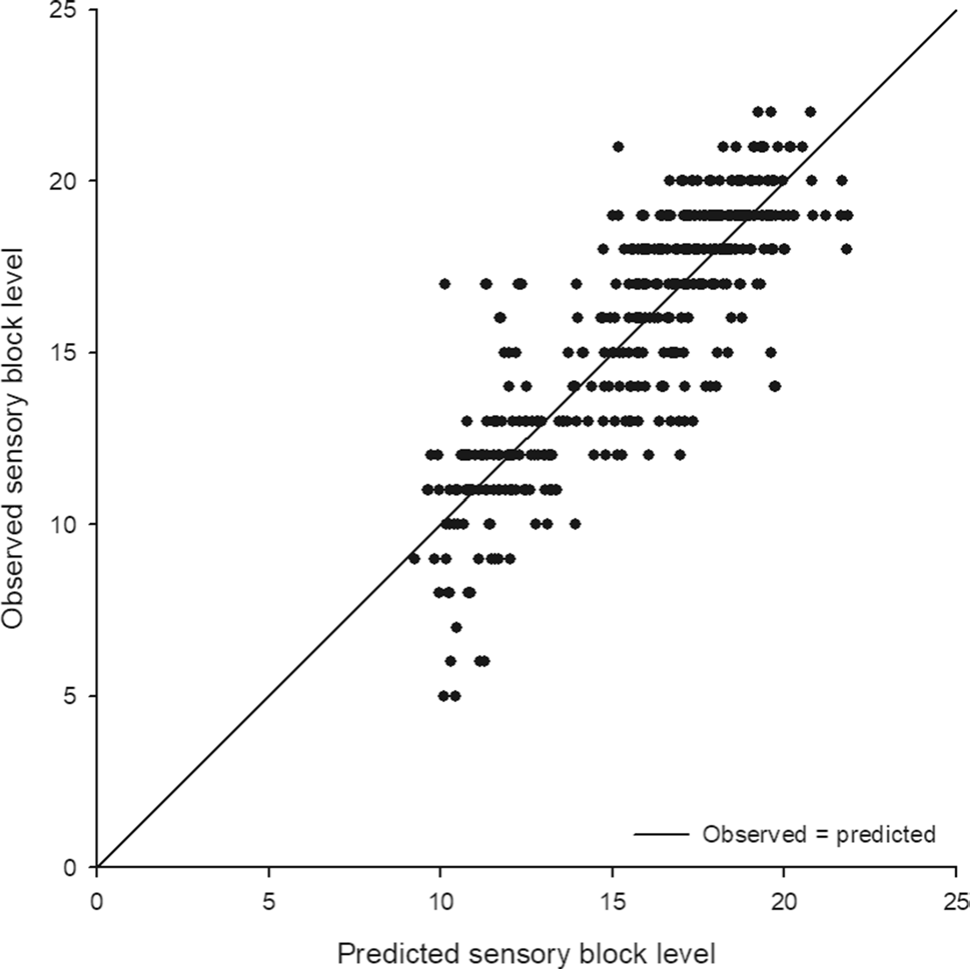
Scatter plot of the predicted and observed sensory block level for the best fit model. The predicted values are calculated using the following formula: 7.12 + 0.88 × (hyperbaric bupivacaine dose in mg) + 1.59 × gender (1 for female, 0 for male) − 0.11 × (height − 162 in cm) + 0.05 × (weight − 68 in kg) + age effect (0.95 for age between 75 and 85; 2.57 for age > 85).

In the multiple regression analysis, five independent predictors of peak block height were identified, including hyperbaric bupivacaine dose, height, weight, sex and age grouping (Table 3). The regression equation between the peak block level and its predictors was Y (peak block level) = 7.12 + 0.88 × (hyperbaric bupivacaine dose in mg) + 1.59 × gender (1 for female, 0 for male) − 0.11 × (height − 162 in cm) + 0.05 × (weight − 68 in kg) + age effect (0.95 for age between 75 and 85; 2.57 for age > 85). The predictive power of the aforementioned model expressed as a coefficient of determination was 0.72. The observed sensory block level was plotted against the predicted block height for the best fit model as Fig. 1. Regarding the interactions between collected variables, no significant interaction effect was found between any two covariates after the multiple regression analysis.

**Table 3:** Selected predictors of peak spinal block level after the model selection.

Characteristic	*β*	SE	Standardized *β*	*p*	*R*^2^	Adjusted *R*^2^
Bupivacaine dosage	0.88	0.03	0.77	< 0.001	0.719	0.715
Height	− 0.11	0.01	− 0.32	< 0.001		
Weight	0.05	0.01	0.21	< 0.001		
Sex (Female vs. male)	1.59	0.25	0.23	< 0.001		
**Age**				< 0.001		
76–85 vs. ≤ 75	0.95	0.27	0.10	< 0.001		
> 85 vs. ≤ 75	2.57	0.44	0.16	< 0.001		
Constant	7.12	0.31		< 0.001		

*β*, regression coefficient; SE, standard error; *R*^2^, coefficients of determination.

## Discussion

How to select a reasonable dose of intrathecal local anaesthetic for the desired block extent in distinct types of surgeries for various patients, is an important clinical issue. We proposed a multiple linear regression model with five common variables which predicted the sensory block height after spinal anaesthesia using hyperbaric bupivacaine with more than 70% predictive power. With the help of this formula, more reliable dose adjustment could be easily implemented.

In this study, we only investigated cases receiving hyperbaric (heavy) bupivacaine instead of plain bupivacaine for spinal anaesthesia for the following reasons. First, the density of plain bupivacaine is close to CSF at room temperature but will become mildly hypobaric after subarachnoid injection at the body’s core temperature of 37 °C^10^. Even a little density change can result in a remarkable variation in intrathecal drug spread^11^. In contrast, heavy solutions remain hyperbaric before and after spinal injection with negligible effects on the intrathecal drug distribution^10^. Second, a procedure effect such as higher levels of injection, may cause more cephalad spread with plain bupivacaine^12–14^ but has little effect on the spread of the heavy solution^15–17^. Since it is difficult to accurately identify the interspace for injection^18^, the use of a hyperbaric solution will minimize the influence of any inaccuracy at the injection site. Third, other procedure related factors, such as the orientation of the needle orifice^19–21^ and the speed of injection cause less influence on block height when using heavy solutions^22–24^. Consequently, we only focused on hyperbaric bupivacaine in spinal anaesthesia due to there being less inter-patient variability and more predictable sensory blockade compared with its isobaric counterpart^25^.

Although a number of patient characteristics may influence intrathecal drug spread, lumbosacral CSF volume acquired from magnetic resonance imaging (MRI) estimation has been postulated as the primary determinant for spinal anaesthetic spread^26^. In clinical practice, it is impractical and unnecessary to obtain the CSF volume data by MRI before spinal anaesthesia. Therefore, our approach provides a quick and useful clinical guide that can be used in daily practice and is based on just five easily available measurements.

It is reasonable to expect that the extent and duration of subarachnoid nerve block depends on the bupivacaine dosage whenever other potentially influential factors are controlled for^27^. With regard to age, previous studies also found that an increased block level could be observed in the elderly^28,29^. It is possible that CSF volume shrinks, and the spinal nerves appear more sensitive to local anaesthetics with advancing age. Notably, our findings support the theory that age was not correlated with block height in a linear manner but became significant beyond the cut off value of 75 years old.

Moreover, we found that gender was also an independent predictor of sensory block height. In the final regression model, women tended to have sensory blocking 1.6 dermatomes higher compared with men after spinal anaesthesia, when the other four explanatory variables were controlled for. Although the mechanism underlying this intersexual disparity is unclear, differences in CSF density may play a role. The movement of subarachnoid local anaesthetics depends on the interaction between the drug and CSF under the influence of gravity. The mean density of CSF is higher in men than in women^30^ and a given intrathecal drug could become less hyperbaric in men and more hyperbaric in women and this could possibly lead to the observed difference in cephalad spread.

In addition, vertebral column length and abdominal girth have recently been reported as newly influencing factors which should replace body height and weight for intrathecal drug spread. Even so, body height and weight are still more easily accessible than measurements of vertebral column length and abdominal girth. Recent studies and our results indicated that block level is negatively correlated with body height whereas it is positively correlated with body weight.

Despite the fact that body weight and age > 85 were not significantly associated with sensory block level after spinal anaesthesia in the univariate analysis (Table 2), in the multivariable analysis, a very significant effect could be demonstrated between sensory block level and both of these variables (Table 3). This resulted from confounding effects between the collected variables and was easily eliminated after the multivariable analysis. For example, females and the elderly were more inclined to have a lower body weight compared with their counterparts and these potential confounding effects could mask the original association between body weight and sensory block height after spinal anaesthesia in the univariate analysis. Therefore, all the collected variables should be evaluated together in the multivariable analysis regardless of the univariate results to avoid analytical bias.

An investigation into the potential interactions between covariates is of practical importance in exploring the influential factors on sensory block level after spinal anaesthesia. In spite of the fact that interaction terms considerably increase the complexity of a predictive model and the difficulty of the explanation and analysis, checking for interactions between collected variables should not be overlooked. However, in the current study we did not identify any interactions between the variables of interest and the combined effects of the five collected variables on sensory block level were roughly additive.

Spinal anaesthesia exhibits differential sensory block to light touch, pinprick and cold temperature discrimination from low to high blocking dermatomes in sequence. We used a soaked alcoholic sponge as the routine method for assessing the patient’s blockage of cold sensations. Although pinprick has long been considered the standard measurement of analgesia representing blockade of A-δ fibres, several studies have also found that block levels to pinprick are very close to those for cold sensation^31–33^. It has been widely suggested that the block level to cold or pinprick testing is considered adequate two to three segments above the expected level of surgical incision^34^.

There were some limitations to the current study. First, although the developed predictive model accounted for over 70% of variations in sensory block level, there were still nearly 30% of unexplained variances which require further investigation. Moreover, other patient characteristics, such as variations in spinal curvature (lordosis, kyphosis and scoliosis), subarachnoid space or CSF volumes are also potential determinants of block level but they were not included in the analysis. In addition, the assumption of equal volume and length in each vertebral space in our model may result in undeveloped bias. Finally, small doses of bupivacaine (< 5 mg) were less frequently used in our study (7%), and so the generalizability of our predictive model beyond the scope of our patient selection is debatable and it should be used with caution.

In conclusion, the current study summarises the association between sensory block level after spinal anaesthesia and five readily available variables in a predictive regression model. This study provides practical and valuable information about the associations between these features and is a useful guide for clinicians to predict sensory block height after single-shot spinal anaesthesia. This could help them to determine the hyperbaric bupivacaine dose with greater ease for various patients who are receiving miscellaneous surgical procedures. The generalizability of our findings requires further investigation and more prospective studies which collect more potentially influential factors are necessary to better predict the sensory block height after spinal anaesthesia with hyperbaric bupivacaine.

## Methods

### Study population and design

This retrospective study was approved by the Institutional Review Board of Cheng-Hsin General Hospital (CHGH-IRB No:(668)107-40) and the need for patient informed consent was waived due to its retrospective design. All research was performed in accordance with relevant guidelines and local regulations. The inclusion criteria were: non-pregnant patients with ASA physical status I-III, aged between 20 and 99 years, and scheduled for surgery on their lower extremities, anorectum or pelvis and lower abdomen between September 2013 and April 2018 under spinal anaesthesia. Those who had neurological deficits, a history of spinal surgery, difficulty in clearly expressing skin sensations or those who had repeated spinal anaesthesia or general anaesthesia conversion were excluded.

All patients were placed in the lateral decubitus position to receive spinal anaesthesia. Following skin disinfection with chlorhexidine, a lumbar puncture was performed by the midline or paramedian approach with a 27-gauge Quincke needle at the L3–4 or L4–5 interspace using the palpated intercristal line technique. Hyperbaric bupivacaine, 0.5% bupivacaine in 8% glucose solution (Marcaine® 0.5% Spinal Heavy, AstraZeneca, France) was used throughout all surgeries. The dosage of hyperbaric bupivacaine for the proposed surgery procedure was determined based on clinical experience and all spinal anaesthetic procedures were performed by the same physician (Y. Y. Huang, an anaesthesiologist with over 10-years’ experience) to reduce potential interindividual variability in the administration of the spinal anaesthesia.

After free flow of clear cerebrospinal fluid (CSF) was obtained, 0.2 ml CSF was aspirated into the syringe for confirmation and then the drug was injected at a speed of about 0.2 ml per second. Patients were turned supine immediately after completion of the intrathecal injection and then sensory testing was conducted by another anaesthetic assistant. Sensory block was defined as loss of cold-temperature sensation by touching the skin with a 75% alcohol-soaked sponge on dermatomes between the bilateral mid-clavicular lines.

The dermatomal block levels were examined at the 2nd and 5th minute after the spinal injection and every 5 min thereafter until the level remained unchanged for three consecutive tests (defined as peak block level). Surgical posture was repositioned after the peak block level had been determined. The block levels of dermatomes (S5, S4, S3, S2, S1, L5…L1_,_ T12…T1; where S: Sacral, L: Lumbar and T: Thoracic) were numerically coded from 1 (S5) to 22 (T1). Electrocardiography (ECG), blood pressure (BP) and oxygen saturation (SpO2) were continuously monitored during the anaesthesia and perioperatively. If the patient’s BP dropped below 30% of their baseline, ephedrine 4–8 mg was titrated intravenously until it returned.

### Statistical analysis

Descriptive statistics were used to describe the patient characteristics. Since the association between age and peak block level (induced loss of cold sensation at the highest dermatome) may not be linear, all patients were further classified into three age groups (age ≤ 75, age > 75 and ≤ 85, and age > 85 years). Simple linear regression analysis was conducted to evaluate the potential correlation between the peak block level and the collected variables (hyperbaric bupivacaine dose, sex, age group, weight and height).

Multiple regression analysis was performed to identify independent predictors of peak block level and to construct the final predictive model. Potential interactions between the collected variables were also checked using hierarchical multiple regression models. The goodness of model fit was evaluated with coefficients of determination (*R*^2^) and adjusted *R*^2^ values. The observed sensory block level was plotted against the predicted block height for the best fit model as well.

According to the suggestion of Tabachnick and Fidell, the minimum number of cases for regression analysis should be more than 40× m, where m is the number of candidate variables in the model^35^. This minimum sample size requirement was met in our analysis. A *p* value < 0.05 was considered to be statistically significant. SPSS software (SPSS Inc., Chicago, IL, USA), version 18.0, was used to analyse the data.
